# The Effect of Discharge Training Based on Teach‐Back Method on Discharge Readiness and Satisfaction: A Randomized Controlled Trial

**DOI:** 10.1111/wvn.70062

**Published:** 2025-07-23

**Authors:** Ayse Gullet, Sevinc Tastan

**Affiliations:** ^1^ Health Sciences Faculty, Nursing Department Eastern Mediterranean University Famagusta, North Cyprus Turkey

**Keywords:** discharge education, discharge readiness, knowledge test, lumbar disc herniation, satisfaction, surgery, teach‐back method

## Abstract

**Background:**

The teach‐back method is an effective approach for reinforcing patient education by clarifying and reviewing misunderstood concepts.

**Aim:**

To examine the effect of discharge training based on the teach‐back method on discharge readiness and satisfaction in patients undergoing lumbar disc herniation surgery.

**Methods:**

A randomized controlled trial using a pre‐test–post‐test design was conducted at two state hospitals in Northern Cyprus from November 2022 to December 2023. A total of 64 patients were randomly assigned to either the intervention group (*n* = 32) or the control group (*n* = 32). Data were collected using the Discharge Education Satisfaction Scale, the Readiness for Hospital Discharge Scale, and the Discharge Education Knowledge Test. The CONSORT 2010 flow diagram was followed.

**Results:**

The mean ages of the intervention and control groups were 51.26 ± 11.92 years and 46.50 ± 11.73 years, respectively. Following the intervention, patients who underwent lumbar disc herniation surgery in the intervention group showed significantly higher scores compared to the control group (*p* < 0.05). These improvements were observed in overall discharge education satisfaction, discharge education knowledge, and all subdimensions of discharge readiness–including personal status, knowledge, and coping ability.

**Linking Evidence to Action:**

Discharge education delivered using the teach‐back method enhances satisfaction, knowledge, and discharge readiness in patients undergoing lumbar disc herniation surgery.

**Trial Registration:** The full research protocol is available at ClinicalTrials.gov (NCT05695014)

## 1 Background

Comprehensive and holistic discharge training, planned according to the patient's needs, helps patients and their relatives adopt positive health behaviors. In addition, it also facilitates their adaptation to the new health condition (Boran and Kose 2023; Marchand et al. 2021). The first days after discharge post‐surgery are a period when patients are vulnerable and need support. After discharge from the hospital, some complications may occur at home. In light of these complications, it is essential that both the patient and their family are well‐informed and adequately prepared (Ahmed Abd‐Ella et al. 2021). Therefore, planned discharge education—an essential component of nursing care—not only helps patients understand their illness and treatment (Ahmed Abd‐Ella et al. 2021; Rizk and Ali 2021), but also increases their participation in the treatment process (Boran and Kose 2023).

Planned discharge training, which is one of the standards of Enhanced Recovery After Surgery in the early postoperative period, positively influences surgical outcomes. It also helps reduce patient anxiety and supports their adaptation to the treatment process (Vinas‐Rios et al. 2018). However, patients often have difficulty understanding or remembering the information given to them. For this reason, different training methods are used to increase the effectiveness of discharge education. One of the methods recommended for use in patient education in recent years is the teach‐back method. The teach‐back method (TBM) is stated to increase patients' knowledge and minimize misunderstandings in health care (Anderson et al. 2020; Oh et al. 2021).

TBM involves asking patients to explain in their own words what information they have received about their treatment means. Thus, any misunderstandings are clarified by the health professional. The patient's understanding of the information is then assessed. This process continues until the patient remembers correctly (Shersher et al. 2021; Yen and Leasure 2019). TBM, which is recommended for addressing misunderstandings in health care and enhancing the effectiveness of discharge education, helps patients cope with their limitations. It also contributes to improving their self‐efficacy. The most basic component of effective health communication is that the provider of education clearly expresses their health status to their patients (Bahri et al. 2018; Ratna 2019). TBM is an effective tool in the process of adaptation to discharge for patients with lumbar disc herniation surgery. Thanks to TBM, the level of knowledge about the diseases of the patients develops, while the rate of re‐hospitalization decreases with the more active participation of post‐discharge patients in their care (Almkuist 2017).

In the literature, it is seen that TBM, which is effective in improving the patient's self‐care, is frequently used, especially in the education of patients with chronic diseases (Chandar et al. 2019; Mollazadeh and Maslakpak 2018; Zabolypour et al. 2020). Effective discharge training provided to patients and their relatives, along with patients' readiness for discharge, makes it possible to maintain optimal home care after surgery. There are a limited number of studies on the use of this method, which has been recommended for use in recent years, in the discharge education of patients who have undergone surgery (Choi and Choi 2021; Ghorbani et al. 2021). Prevention of complications after lumbar disc herniation surgery and acceleration of recovery is possible with effective discharge training. Although the importance of patient education is known, many studies emphasize that patients' education is not given enough importance and returns to the hospital after discharge increases (Ghorbani et al. 2021). Getting planned discharge training for patients who undergo lumbar disc herniation (LDH) surgery not only helps to improve their physical function after surgery but also its therapeutic effects, reducing pain, increasing self‐care power, increasing quality of life, and reducing costs (Quan 2022; Sınmaz and Akansel 2021). In this study, it was aimed to examine the effect of the discharge training given by the method of teaching back to LDH surgery patients on the satisfaction of the patients regarding the readiness for discharge and the discharge training. It is thought that the results of this study, which will reveal the effect of TBM on the satisfaction of the patients with the discharge training and their readiness for discharge, will contribute to the transfer of current approaches to patient education into practice. The primary purpose of this study was to examine the effect of discharge training delivered via TBM on LDH surgery patients' discharge satisfaction and readiness. The secondary purpose of the study is to examine the effect of discharge education provided to patients using the TBM on their discharge‐related knowledge. The study included two groups of patients: one that received discharge training using the Teach‐Back Method, and one that received standard discharge training. The hypotheses of the study were as follows:

*There is no significant difference in discharge readiness scores between the two groups*.

*There is no significant difference in discharge education satisfaction scores between the two groups*.

*There is no significant difference in discharge education knowledge test scores between the two groups*.

## 2 Methods

### 2.1 Study Design and Setting

This study was conducted as a pre‐test, final test randomized trial with a control group. The research was carried out in the Neurosurgery clinics of two state hospitals in Northern Cyprus between November 2022 and December 2023. The study followed CONSORT 2010 Guidelines (Supporting Information).

### 2.2 Sampling

The sample size, significance level, and effect size were determined using G Power 3.1.9.4. The sample needed was at least 68 people, 34 in each group, under the assumptions of a 95% confidence interval, 80% power, and 0.50 effect size. In cases where the significance level was not specified in the study, the significance level *a* = 0.05 was used. In addition, the effect size was taken to be 0.50. The study included patients aged 18 and over who underwent elective microdiscectomy surgery. Participants were required to be able to read and understand the research guidelines. Patients who did not agree to participate in the study and who underwent emergency surgical interventions were not included.

### 2.3 Randomization

**FIGURE 1:**
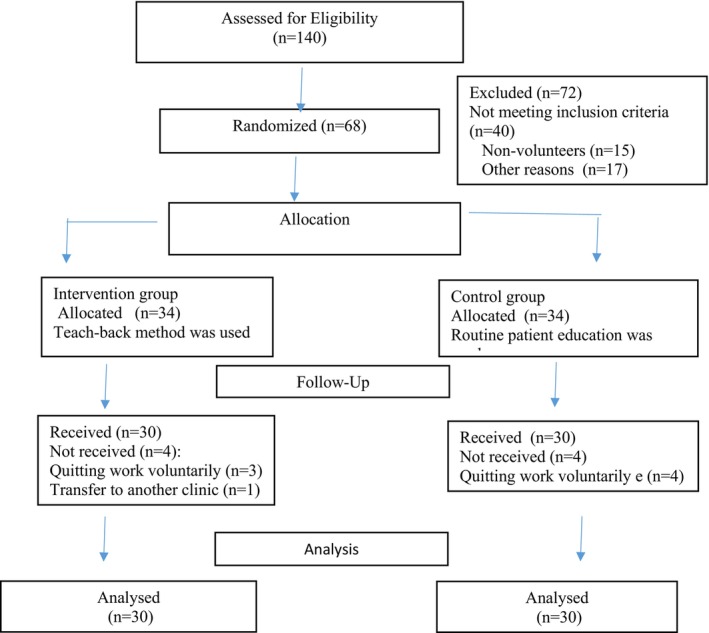
Flowchart of the progress of individuals in the phases of the controlled clinical trial. CONSORT 2010 flow diagram.

Patients were randomized into a study group using random allocation software (Random Alloc, Ver. 2.0.0). Sixty patients were randomized (30 to the Intervention Group, 30 to the Control Group) (Figure 1).

### 2.4 Outcomes

The main outcome of this study were satisfaction and readiness for discharge of patients who underwent lumbar disc herniation surgery. Patients' satisfaction levels were measured with the Discharge Training Satisfaction Scale, and their readiness for discharge was measured with the Readiness for Hospital Discharge Scale. A secondary outcome was to examine the effect of TBM used in patient education on discharge information. The discharge education knowledge of the patients was measured with the Discharge Education Knowledge Test.

### 2.5 Data Collection Tools

A descriptive information form, Discharge Training Satisfaction Scale, Readiness for Hospital Discharge Scale, and Discharge Training Knowledge Test were used for data collection.

#### 2.5.1 Descriptive Information Form

The form included socio‐demographic characteristics such as date of birth, gender, marital status, level of education, and substances used related to the patient's disease.

#### 2.5.2 Discharge Training Satisfaction Scale

The scale was developed by Oak and Köşgeroğlu in 2021 (Meşe and Köşgeroğlu 2021). The scale consists of 21 items. The Cronbach α reliability coefficient of the scale overall was found to be 0.91. Item responses use a 5‐point Likert scale: 5 = *I am completely satisfied*, 4 = *I am satisfied*, 3 = *I am partially satisfied*, 2 = *I am not satisfied*, and 1 = *I am not satisfied at all*. Total scores vary between 21 and 105. The scale has no breakpoint.

#### 2.5.3 Readiness for Hospital Discharge Scale

The Readiness for Hospital Discharge Scale consists of 8 items and 4 dimensions. Each item is rated on a scale from 0 to 10, with higher scores indicating greater readiness for discharge (Weiss et al. 2014). The scale consists of four dimensions: personal status (items 1 and 2), knowledge (items 3 and 4), coping ability (items 5 and 6), and expected support (items 7 and 8). The first 2 items evaluate how individuals feel throughout the day. The next two items assess their level of knowledge about discharge. Items 5 and 6 evaluate how and in what way they can continue their daily lives at home after discharge. Finally, the last two items assess the support they can receive at home after discharge. In the Turkish version of the scale, the Cronbach α reliability coefficient was found to be 0.74 for the general scale, and on the subscales, this value was found to be 0.79–0.93 (Kaya et al. 2018).

#### 2.5.4 Discharge Training Knowledge Test

In the prepared knowledge test, there were 20 questions containing right and wrong answers. Patients were asked to choose their answers from the options: Right, False, and No Idea. In the evaluation of the knowledge test results, the correct answer is scored as 1. Both the incorrect and no idea responses are scored as 0. The score range of the knowledge test varies between 0 and 20 points. Higher scores indicate higher patient knowledge levels, and lower scores indicate lower patient knowledge. Expert opinion was received from 9 nursing academics working in the field to ensure content validity (Polit and Beck 2021). Two of these nurse academics had expertise in surgical nursing, specifically in the neurosurgery service. In addition, two academic nurses had previous experience with LDH surgery. The specialties of others were public health nursing. The content validity index (CVI) values of the knowledge test were higher than 0.80. Before the main study, a pilot test of the knowledge test was conducted with a group of 10 patients to confirm that the items were understandable and appropriate for the target population. The original version of the knowledge test was developed and validated in Turkish. The English version presented in this article was translated for publication purposes and was not used in data collection.

### 2.6 Interventions

Upon hospitalization, patients who met the inclusion criteria for the study were approached in their rooms, where written and verbal informed consent was obtained. Following this, participants were assigned to either the control or intervention group based on a randomization table, or the data collection process commenced.

For patients in the control group, the Descriptive Information Form, the Readiness for Hospital Discharge Scale, and the Discharge Training Knowledge Test were administered at baseline. Discharge education for these patients was provided by the healthcare professionals responsible for their care, following standard clinical protocols. On the day of discharge, the Readiness for Hospital Discharge Scale and Discharge Training Knowledge Test were re‐administered as post‐tests, followed by the completion of the Discharge Training Satisfaction Scale.

Patients in the intervention group completed the Descriptive Information Form, the Readiness for Hospital Discharge Scale, and the Discharge Training Knowledge Test during their admission to the clinic. A suitable time for discharge training was then scheduled, typically the evening before the patient's surgery. The discharge training was delivered using TBM, with an average duration of 30 min. During the training, patients were encouraged to repeat key information to confirm their understanding. Additionally, one or two questions related to each sub‐topic in the training booklet were posed to the patients to ensure comprehension. Any incorrect or incomplete information was clarified until the patient demonstrated confidence in their understanding.

The training material used for the discharge education was the Discharge Training Booklet, developed by the researcher based on a comprehensive literature review. This booklet provided detailed information on post‐operative care following LDH surgery. It covered topics such as the planned surgical procedure, wound care, pain management, follow‐up appointments, weight management, nutrition, exercise, and travel restrictions. Additionally, it included information on potential complications and their management, indications for consulting a physician, bathing guidelines, the importance of proper body mechanics, sexual activity, as well as the expected timeline for returning to work (Duojun et al. 2021; Elsharkawy et al. 2018; Lewandrowski and Yeung 2020; Sınmaz and Akansel 2021). The content validity of the booklet was assessed using CVI, as evaluated by an expert panel (Polit and Beck 2021), with a CVI score exceeding 0.80, confirming its validity. On the day of discharge, the Readiness for Hospital Discharge Scale and the Discharge Training Knowledge Test were re‐administered as post‐tests. Afterward, the Discharge Training Satisfaction Scale was completed, concluding the data collection process. To avoid influencing the data collection process, the booklet was provided to patients in the control group only after the completion of the post‐tests on the day of discharge, thereby ensuring ethical responsibilities were upheld.

### 2.7 Statistical Analysis

Statistical Package for Social Sciences 24.0 statistical data analysis software was used in the statistical analyses of the data. The distributions of the introductory characteristics were shown in the frequency distribution tables. The Kolmogorov–Smirnov test was used to assess whether the patients involved in this study showed normal distribution. The Wilcoxon signed‐rank test was used to compare the pre‐test and post‐test scores within groups. The Mann–Whitney *U* test, a non‐parametric alternative to the independent samples *t*‐test, was used to compare the intervention and control groups.

### 2.8 Ethical Issues

To carry out the research, ethics committee permits were obtained from the university where the researchers worked (Date: 23 November 2022; Ethics Approval Number: ETK00‐2022‐0262) and the State Hospital where the study was carried out (Date: 28 October 2022; Ethics Approval Number: YTK.1.01 EK 47/22). Written permission was obtained from the patients participating in the research with the ‘Voluntary Informed Consent Form’. Necessary permissions were obtained by e‐mail from the authors of the scales used in the research.

## 3 Results

As seen in Table 1, some introductory characteristics of the patients who underwent LDH surgery were compared between the intervention and control groups. A cross‐tabulation and chi‐squared test were used to assess group homogeneity. According to the results, the intervention and control groups did not differ significantly in age, LDH levels, gender, chronic diseases, employment, or exercise habits. VAS scores for low back and leg pain were also similar between the groups (*p* > 0.05). These findings indicate that the groups were homogeneous (*p* > 0.05). A significant difference was found between the post‐test scores of the Discharge Training Satisfaction Scale (*z* = −6.66; *p* < 0.001) in patients who underwent LDH surgery, constituting the intervention and control groups, as shown in Table 2, in favor of the intervention group (*p* < 0.05).

**TABLE 1:** Baseline demographic and clinical characteristics for intervention and control groups.

Variable	Intervention group (*n* = 30)		Control group (*n* = 30)		*χ* ^2^	*p*
	*n*	%	*n*	%		
Age years					0.617	0.432
45 years and under	11	36.7	14	46.7		
46 years and above	19	63.3	16	53.3		
Level of LDH					2.43	0.487
L2–L3	5	16.7	2	6.7		
L3–L4	11	36.7	16	53.3		
L4–L5	10	33.3	8	26.7		
L5–S1	4	13.3	4	13.3		
Sex					0.067	0.796
Female	15	50.0	14	46.7		
Male	15	50.0	16	53.3		
Presence of chronic diseases					0.617	0.432
No	16	53.3	19	63.3		
Yes	14	46.7	11	36.7		
Working status						
No	14	46.7	9	30.0	1.76	0.184
Yes	16	53.3	21	70.0		
Exercise status						
No	25	83.3	21	70.0	1.49	0.222
Yes	5	16.7	9	30.0		
Low back pain levels groups						
Mild	1	3.3	—	—	2.06	0.355
Moderate	1	3.3	—	—		
Severe	28	93.3	30	100.0		
Leg pain levels groups						
Mild	1	3.3	—	—	2.87	0.238
Moderate	1	3.3	4	13.3		
Severe	28	93.3	26	86.7		

Abbreviation: *χ* ^2^, chi‐squared.

**TABLE 2:** Comparison of post‐test scores on the patients' discharge training satisfaction scale.

Variable	Intervention Group (*n* = 30)		Control Group (*n* = 30)		Test^a^
	M ± SD	Median (min–max)	M ± SD	Median (min–max)	
Discharge training satisfaction scale total	100.70 ± 3.48	101.50 (92–105)	59.86 ± 11.8	61.00 (27–81)	*Z* = −6.66 *p* < 0.001

Abbreviations: M, mean; Max, maximum; Min, minimum; SD, standard deviation.

^a^ Mann Whitney *U* test.

As shown in Table 3, no significant difference was found between the pretest scores of the discharge training knowledge test (*z* = −1.37; *p* = 0.171) of LDH patients who formed the intervention and control groups (*p* > 0.05). A significant difference was found between the posttest scores of the discharge training knowledge test (*z* = 6.76; *p* < 0.001) of LDH patients who formed the intervention and control groups in favor of the intervention group (*p* < 0.05).

**TABLE 3:** Comparison of patients' discharge training knowledge test pretest and posttest scores.

Variable	Intervention Group		Control Group		Test^a^
	M ± SD	Median (min–max)	M ± SD	Median (min–max)	
*Pretest*					
Discharge training knowledge test	7.47 ± 3.12	7.00 (3–18)	8.10 ± 2.44	8.00 (5–16)	*Z*: −1.37 *p* = 0.171
*Posttest*					
Discharge training knowledge test	19.47 ± 0.73	20.00 (18–20)	11.13 ± 2.58	10.5 (7–17)	*Z*: −6.76 *p* < 0.001
Test^b^	*Z*: −4.71	*p* < 0.001	*Z*: −4.13	*p* < 0.001	

Abbreviations: M, mean; Max, maximum; Min, minimum; SD, standard deviation.

^a^ Mann Whitney *U* test.

^b^ Wilcoxon signed‐rank test.

Table 4 presents the comparison results of the pretest and posttest scores on the Readiness for Hospital Discharge Scale. The analysis includes both the overall scores and sub‐dimension scores for patients in the intervention and control groups. Accordingly, there was no significant difference between the intervention and control groups in terms of the pretest scores of the overall scale (*z* = −0.126; *p* = 0.900). Similarly, no significant differences were found in the sub‐dimensions: personal status (*z* = −0.566; *p* = 0.571), knowledge (*z* = −0.350; *p* = 0.726), coping ability (*z* = −0.690; *p* = 0.490), and expected support (*z* = −0.128; *p* = 0.898) (*p* > 0.05). After the intervention, a significant difference was found in favor of the intervention group. This difference was observed in the overall scale score (*z* = −5.15; *p* < 0.001), as well as in the sub‐dimensions of personal status (*z* = −3.32; *p* < 0.001), knowledge (*z* = −6.44; *p* < 0.001), and coping ability (*z* = −2.87; *p* = 0.004). No statistically significant difference was found between the posttest scores of the patients' expected support (*z* = −1.37; *p* = 0.170) sub‐dimension of the Readiness for Hospital Discharge Scale of the patients in the intervention and control groups (*p* < 0.05).

**TABLE 4:** Comparison of patients' readiness for hospital discharge scale general and sub‐dimension pretest and posttest scores.

Readiness for hospital discharge scale	Intervention		Control		Test^a^	*p*
	M ± SD	Median (min–max)	M ± SD	Median (min–max)		
*Pretest*						
Total scale	*41.43 ± 13.61*	*44.00 (17–73)*	*41.07 ± 12.45*	*43.50 (18–63)*	*Z*: −0.126	0.900
The patient's personal status	7.83 ± 3.73	8.00 (2–18)	7.17 ± 2.83	7.00 (2–14)	*Z*: −0.566	0.571
The patient's knowledge	6.50 ± 4.67	6.00 (0.00–20)	5.77 ± 3.21	5.50 (0.00–16)	*Z*: −0.350	0.726
The patient's coping ability	11.27 ± 4.06	12.00 (5–18)	11.97 ± 4.81	13.5 (3–19)	*Z*: −0.690	0.490
The patient's expected support	15.83 ± 4.53	18.00 (6–20)	16.17 ± 4.28	17.00 (5–20)	*Z*: −0.128	0.898
*Posttest*						
Total scale	70.07 ± 7.08	73.00 (52–79)	53.73 ± 13.68	58.50 (17–74)	−5.15	< 0.001
The patient's personal status	*15.60 ± 2.03*	*16.00 (11–19)*	*12.40 ± 3.72*	*13.00 (6–17)*	−3.32	0.001
The patient's knowledge	*18.27 ± 2.02*	*19.00 (13–20)*	*9.70 ± 3.00*	*10.00 (3–18)*	−6.44	< 0.001
The patient's coping ability	17.67 ± 2.09	18.00 (13–20)	14.63 ± 4.63	17.00 (3–19)	−2.87	0.004
The patient's expected support	18.53 ± 2.05	20.00 (13–20)	17.00 ± 4.15	18.00 (4–20)	−1.37	0.170

Abbreviations: M, mean; Max, maximum; Min, minimum; SD, standard deviation.

^a^ Mann Whitney *U* test.

## 4 Discussion

This research was carried out to examine the effect of discharge training, which was given by TBM to patients after LDH surgery, on the satisfaction levels of readiness for discharge and discharge training. In this study, the patients in the intervention and control groups were found to be similar in terms of age distribution, LDH levels, gender, presence of chronic disease, employment status, regular exercise habits, and VAS scores for low back and leg pain. The postoperative process is a great source of stress for the patient and needs to be well managed. Good management of the postoperative process and increasing the satisfaction of patients can be achieved with well‐planned discharge training (Arslan and Gürsoy 2021). In this study, it was seen that the discharge education satisfaction scale scores of the patients in the intervention group were higher than those in the control group (*p* < 0.05). We can say that the reason for the difference in the mean score between the groups is due to the different method used in discharge training. In this study, discharge education was given to the intervention group using TBM, and the patient education booklet prepared by the researchers was used in the education of the patients. Patients in the control group, on the other hand, received their discharge training from their clinical doctor and nurse, as in routine practices. In this study, similar to the literature (Shersher et al. 2021; Talevski et al. 2020), we can say that the discharge training given with TBM increases patient satisfaction more than the discharge training given according to normal clinical routines. Studies also indicate that TBM enhances communication between healthcare personnel and patients, and contributes positively to the discharge process (Shersher et al. 2021; Talevski et al. 2020).

The process after LDH surgery can only be managed with good discharge planning. In this study, no significant difference was found between the LDH discharge training information test pretest scores of the patients in the intervention and control groups. In the study, when intra‐group comparisons were examined, it was seen that the scores of the patients in the intervention and control groups increased after the discharge training. However, when comparisons between groups were examined, it was seen that the discharge information test score of the intervention group was higher. According to these results, the discharge training given to the intervention group by TBM positively affected the total score of the LDH discharge training knowledge test. TBM is one of the current methods used to ensure the good transfer of discharge education to patients and the permanence of education (Çatal and Cebeci 2023; Zhang et al. 2023). Similar to the results obtained from this study, many research results show that TBM positively improves readiness for discharge (Oh et al. 2021; Yen and Leasure 2019). In a meta‐analysis conducted by Yen and Leasure (2019), it was concluded that the teach‐back method is effective in increasing patients' knowledge about their disease and promoting active participation in their treatment. It was also found to be a safe tool for improving patient satisfaction, as it has no known negative consequences (Yen and Leasure 2019). In addition, findings in other studies have demonstrated that TBM has an important place in facilitating the adaptation of patients to the situation by increasing self‐control in chronic diseases, as well as in discharge training (Hemamali et al. 2024; Oh et al. 2023; Talevski et al. 2020). Although surgery improves the symptoms caused by the disease in individuals, it still requires individuals to make changes in their current routine. The surgery itself is a source of psychological as well as physical stress for the individual, and individuals may not fully grasp what is said during patient education. In this case, using TBM is a good option. During TBM, it becomes possible to assess what the patient understands and to what extent, and to distinguish between correct and incorrect information. Accordingly, the training is repeated. The training continues to repeat until it is clear that the patient has received the correct information. In this context, it can be said that TBM discharge training is an effective and positive teaching method for increasing the knowledge level of the patients and preparing them for discharge.

Good preparation for discharge will accelerate the healing process by ensuring that the patient adapts to the existing situation in the early period (Burucu et al. 2023; Durmaz and Özbaş 2023). In this study, it was observed that discharge training provided to the intervention group using TBM had a positive effect on the overall score of the Readiness for Hospital Discharge Scale, as well as on the sub‐dimensions of personal status, knowledge, and coping ability. The training effectively enhanced patients' readiness for discharge. In the study by Oh et al. (2023), the effectiveness of discharge training using TBM was evaluated in patients with heart failure. The results showed that this method increased patients' knowledge levels, improved their discharge readiness, and ultimately helped reduce hospital readmissions. It is thought that the use of TBM by healthcare professionals in post‐surgical patient education will facilitate the adaptation of their patients to their post‐operative lives and provide early recovery.

### 4.1 Implications for Future Research

The use of TBM in routine discharge training, which increases patient satisfaction, should be encouraged and supported by healthcare professionals. By improving patients' discharge knowledge and readiness, TBM can support active patient participation in care and contribute to positive health outcomes.

### 4.2 Limitations

This study has two limitations. First, the study only included patients from two state hospitals in Northern Cyprus who underwent LDH surgery, so the generalizability of the results may be limited. Second, the patients participating in the study responded to the study's measurement tools according to their perceptions.

### 4.3 Linking Evidence to Action

- Discharge training based on TBM effectively improved discharge readiness in patients who underwent LDH surgery.
- Discharge training programs by TBM helped increase patient satisfaction.
- Using TBM significantly increased the discharge knowledge of patients.
- TBM in the discharge training of patients improved patient care outcomes.

## 5 Conclusion

The results of this study indicated that TBM delivered to patients who underwent LDH surgery positively influenced their satisfaction, discharge‐related knowledge, and readiness for discharge. Therefore, it is very important to inform and encourage clinical nurses to use TBM in the discharge training of other patient groups undergoing different types of surgery. The use of TBM in routine discharge training, which increases patient satisfaction, should be encouraged and supported by healthcare professionals. TBM can enhance patients' discharge knowledge and readiness, thereby promoting active involvement in their care, which in turn contributes to improved health outcomes. For future research, it is recommended to include patients' relatives during TBM to evaluate the results and to examine the long‐term effect of this method on patient outcomes. Other suggestions include conducting the study in different larger sample groups and multicenters. In addition, including a cost analysis and examining whether TBM implementation reduces 30‐day readmission rates would also strengthen the evidence base.

## Conflicts of Interest

The authors declare no conflicts of interest.

## Supporting information


**Data S1:** wvn70062‐sup‐0001‐supinfo.docx.

## Data Availability

The data that support the findings of this study are available on request from the corresponding author. The data are not publicly available due to privacy or ethical restrictions.
